# Carbon nanohorn-based nanofluids: characterization of the spectral scattering albedo

**DOI:** 10.1186/1556-276X-7-96

**Published:** 2012-02-01

**Authors:** Luca Mercatelli, Elisa Sani, Annalisa Giannini, Paola Di Ninni, Fabrizio Martelli, Giovanni Zaccanti

**Affiliations:** 1 National Research Council-National Institute of Optics (CNR-INO), Largo E. Fermi, 6, Florence, 50125, Italy; 2 Department of Physics and Astronomy, University of Florence, via Sansone 1, Sesto Fiorentino, 50019, Italy

## Abstract

The full characterization of the optical properties of nanofluids consisting of single-wall carbon nanohorns of different morphologies in aqueous suspensions is carried out using a novel spectrophotometric technique. Information on the nanofluid scattering and absorption spectral characteristics is obtained by analyzing the data within the single scattering theory and validating the method by comparison with previous monochromatic measurements performed with a different technique. The high absorption coefficient measured joint to the very low scattering albedo opens promising application perspectives for single-wall carbon nanohorn-based fluid or solid suspensions. The proposed approximate approach can be extended also to other low-scattering turbid media.

**PACS: **78.35.+c Brillouin and Rayleigh scattering, other light scattering; 78.40.Ri absorption and reflection spectra, fullerenes and related materials; 81.05.U- carbon/carbon-based materials; 78.67.Bf optical properties of low-dimensional, mesoscopic, and nanoscale materials and structures, nanocrystals, nanoparticles, and nanoclusters.

## Introduction

Single-wall carbon nanohorns [SWCNHs] are carbon nanostructures belonging to the family of carbon nanotubes. They consist of single layers of a graphene sheet wrapped into an irregular tubule with a variable diameter of 2 to 5 nm and a length of 30 to 50 nm, with cone-shaped tips [1-3]. The SWCNHs assemble to form roughly spherical aggregates with typical diameters of about 100 to 120 nm with three characteristic morphologies: dahlia-like, bud-like, and seed-like [3]. They exhibit both a large surface area and a large number of cavities [4] and therefore appear promising for a large variety of applications, including gas storage [5], drug delivery, [6] and solar energy [7,8]. When the differences between SWCNHs and better-known carbon nanotubes are concerned, the absence of metals in SWCNHs (which are needed to catalyze nanotube growth) makes their cytotoxicity negligible [9]. Moreover, the minimum van der Waals interactions between the superstructures of SWCNH aggregates give rise to a better dispersion of SWCNHs in liquid media [10] and a much longer time stability of their suspensions. In fact, SWCNH aqueous suspensions have been demonstrated to be very stable [7] also when compared to more conventional carbon forms like amorphous carbon [11]. Recently, we studied SWCNH-based nanofluids, proposing them as direct absorber and heat exchange medium for solar collector applications [9], and we measured the scattering albedo at some Vis-near infrared [NIR] wavelengths [12]. The low albedo values measured could open interesting perspectives of applications for this kind of nanomaterial also in other different fields.

In the present paper, we propose a spectrophotometric method for the spectral evaluation of the scattering albedo of SWCNH aqueous suspension. The results obtained with the proposed method have been compared to those recently obtained at some discrete wavelengths using a different technique [8,12], showing a fair agreement.

## Materials and methods

SWCNHs were produced with a patented method [13], able to selectively produce different morphologies of SWCNHs (dahlia-like, bud-like, and seed-like). Some dispersant is necessary to avoid aggregation of nanoparticles in water, and sodium *n*-dodecyl sulfate (99%, Alfa Aesar, Ward Hill, MA, USA) was demonstrated to be the best dispersant for this kind of carbon nanostructure [14]. For the present work, we used two SWCNH suspensions, with dahlia- and bud-like nanohorn morphologies, labeled in the following as D and B, respectively. Both suspensions had the same nanoparticle concentration (0.3 g/l) and the same surfactant concentration (1.8 g/l).

The spectral scattering albedo has been obtained from spectrophotometric measurements carried out by means of a double-beam spectrophotometer (Lambda 900, PerkinElmer, Waltham, MA, USA) equipped with an integrating sphere for the measurement of transmittance (∅ = 150 mm, radius of the input aperture: *R *= 9.5 mm). A specially designed sample cell was manufactured. The cell dimensions (surface, 95 × 40 mm^2^; thickness, *L *= 5 mm) were chosen in such a way to provide enough internal volume to allow several additions of SWCNH suspensions to pure water and to provide low noise curves with several data points for the chosen experimental method (see below).

The method we propose consists of measuring the transmittance for different concentrations of SWCNH (six progressive additions to pure water of a known amount of the original undiluted concentration). For each concentration, the measurement is repeated with the cell at two different distances from the integrating sphere: with the cell 'far' at a distance (*d*_far _= 160 mm) and with the cell 'near' the integrating sphere, in contact with the aperture (*d*_near _= 0 mm). The two measurements differ for the different fractions of scattered received power. The scattering albedo is obtained from these measurements, making the assumption that the SWCNH particles are sufficiently small with respect to the wavelength, so their scattering function can be approximated with the Rayleigh scattering function.

**Figure 1:**
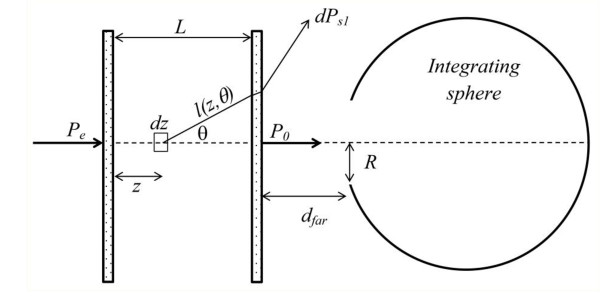
**Geometry used for the model**.

The expression for the scattering albedo has been obtained starting from the power *P*_R _received by the integrating sphere, which is given by:

(1) $$P_{\text{R}}=P_{\text{0}}+P_{\text{S}},$$

where *P*_0 _is the ballistic component, and *P*_S _is the fraction of scattered power that enters the integrating sphere. With reference to Figure 1, *P*_0 _is related to the impinging power *P_e _*by:

(2) $$P_{0}\;=\;T\left(\theta =0\right)P_{e}e^{-\mu_{e}L},$$

where *T*(*θ = *0) is the transmittance of the cell windows (that takes into account the losses due to Fresnel reflections for normal incidence), and *μ*_e _is the extinction coefficient. We remind that *μ*_e _is the sum of the scattering (*μ*_s_) and absorption (*μ*_a_) coefficients, and the scattering albedo *ω *is defined as the ratio *ω = μ*_s_*/μ*_e_. The extinction coefficient, being proportional to the particle concentration, can be expressed as *μ*_e _*= ε*_e_*ρ*, where *ρ *is the concentration of SWCNH particles (in grams per liter) and *ε*_e_, their specific extinction coefficient (per millimeter per (gram per liter)).

Measurements have been carried out for moderate values of the optical thickness *τ*_e _*= μ*_e_*L *(< 2.5). For these values of *τ*_e _and for the low values expected for the scattering albedo (*ω *< 0.1), the scattered power is dominated by the contribution *P*_S1 _due to single scattering, so *P*_S _≅ *P*_S1_. *P*_S1 _is given by [15]:

(3) $$P_{\text{S1}}\;=\;P_{\text{e}}\overset{L}{\underset{0}{\int }}e^{-\mu_{\text{e}}z}\omega \mu_{\text{e}}\overset{\alpha }{\underset{0}{\int }}2\pi p\left(\theta \right)\text{sin}\theta T\left(\theta \right)e^{-\mu_{\text{e}}l\left(z,\theta \right)}d\theta dz=P_{0}\mu_{\text{e}}L\omega ℑ\left(\alpha \right),$$

where *p(θ) *is the scattering function, *l(z,θ) = (L-z)/*cos*θ*, and

(4) $$ℑ\left(\alpha \right)=\frac{1}{L}\overset{L}{\underset{0}{\int }}\overset{\alpha }{\underset{0}{\int }}2\pi p\left(\theta \right)\text{sin}\theta \frac{T\left(\theta \right)}{T\left(\theta =0\right)}e^{-\mu_{e}\left[\left(L-z\right)\left(\frac{1}{\text{cos}\theta }-1\right)\right]}d\theta dz,$$

The angle *α *is the largest value of *θ *for which photons can be detected after a single scattering event. It is determined by total reflection at the water-glass-air interface, and its values are *α*_near _*= *48.7° for the near position and *α*_far _*= *2.55° for the far one.

If it is possible to assume that $e^{-\mu_{e}\left(L-z\right)/\text{cos}\theta }≅e^{-\mu_{e}\left(L-z\right)}$, then ℑ(*α*) becomes independent on *μ*_e _and consequently on the concentration *ρ*. This approximation means that the attenuation after a scattering event at point *z *on the optical axis, due to the path in the cell that exceeds the remaining path *(L-z)*, can be disregarded. This hypothesis will be discussed in more detail below. Under this approximation, ℑ(*α*) becomes:

(5) $$ℑ\left(\alpha \right)≅\frac{1}{L}\overset{\alpha }{\underset{0}{\int }}2\pi p\left(\theta \right)\text{sin}\theta T\left(\theta \right)/T\left(\theta =0\right)d\theta .$$

The power received for a *ρ *concentration of SWCNH can be written as:

(6) $$P_{\text{R}}\left(\rho ,\alpha \right)≅T\left(\theta =0\right)P_{\text{e}}e^{-\varepsilon_{\text{e}}\rho L}\left[1+\varepsilon_{\text{e}}\rho L\omega ℑ\left(\alpha \right)\right],$$

and being $\varepsilon_{\text{e}}\rho L\omega ℑ\left(\alpha \right)<<\text{1}$ (low scattering regime and albedo < 0.1), we have

(7) $$\text{ln}P_{\text{R}}\left(\rho ,\alpha \right)\;≅\;-\varepsilon_{\text{e}}\rho L\left[1-\omega ℑ\left(\alpha \right)\right]+\text{ln}\left[T\left(\theta =0\right)P_{\text{e}}\right].$$

We measured the sample transmittance at six different concentrations. From Equation 7, it is possible to obtain the measured intrinsic extinction coefficient *ε*_e meas _from the slope of ln*P*_R_*(ρ,α) *as a function of *ρ*:

(8) $$\varepsilon_{\text{e meas}}\left(\alpha \right)=\varepsilon_{\text{e}}\left[1-\omega ℑ\left(\alpha \right)\right].$$

Finally, the albedo can be obtained as:

(9) $$\omega \;=\;\frac{\;\varepsilon_{\text{e meas}}\left(\alpha_{\text{far}}\right)-\varepsilon_{\text{e meas}}\left(\alpha_{\text{near}}\right)}{\varepsilon_{\text{e}}\left[ℑ\left(\alpha_{\text{near}}\right)-ℑ\left(\alpha_{\text{far}}\right)\right]}\;≅\;\frac{\;\varepsilon_{\text{e meas}}\left(\alpha_{\text{far}}\right)-\varepsilon_{\text{e meas}}\left(\alpha_{\text{near}}\right)}{\varepsilon_{\text{e meas}}\left(\alpha_{\text{far}}\right)\left[ℑ\left(\alpha_{\text{near}}\right)-ℑ\left(\alpha_{\text{far}}\right)\right]},$$

where we assumed that *ε*_e _≅ *ε*_e means_(*α*_far_). To obtain *ω*, it is therefore necessary to assume a model for the scattering function in order to evaluate ℑ*(α)*. As mentioned before, for the SWCNH particles, we considered the Rayleigh scattering function $p\left(\theta \right)\;=\;\left(\frac{3}{16\pi }\right)\left[1\;+\text{co}{\text{s}}^{2}\theta \right]$.

Equation 9 has been obtained, making some assumptions that need to be summarized and discussed. They, enumerated in order of importance, are (1) the Rayleigh scattering function for the SWCNH particles, (2) ℑ*(α) *independent on the extinction coefficient (Equation 5), (3) *P*_S _≅*P*_S1_, (4) negligible effect of internal reflections, (5) $\varepsilon_{\text{e}}\rho L\omega ℑ\left(\alpha \right)<<\text{1}$, and (6) *ε*_e _≅ *ε*_e means_(*α*_far_).

As for hypothesis (1), the Rayleigh scattering is probably not strictly applicable to SWCNHs especially at short wavelengths. Anyway, it should be noticed that the Rayleigh scattering function is nearly isotropic, while different scattering functions become more and more forward-peaked as the size of particles increases. Therefore, the value of ℑ*(α) *we obtained in hypothesis (1) represents the lower limit, and the resulting value of the scattering albedo can be overestimated, thus representing a higher limit for the albedo itself. To evaluate the error due to this approximation, we calculated ℑ*(α) *using the Mie scattering function for a graphite sphere with a diameter of 100 nm immersed in water. The values we obtained in this case both for ℑ*(α*_far_*) *and ℑ*(α*_near_*) *were 74%, 20%, and 9% higher than those of the Rayleigh scattering at *λ *= 350, 600, and 850 nm, respectively. However, it should be noticed that actual nanoparticles, being aggregates of individual SWCNHs, are strongly nonhomogeneous, and the Mie theory neither is strictly applicable. Their morphology could suggest, as for the light-particle interaction, a sort of effective radius, smaller than the physical radius.

Assumption (2) has been evaluated comparing the approximated values of ℑ*(α) *obtained from Equation 5 with the exact values obtained from Equation 4. Values calculated with Equation 5 are independent on *μ*_e _and equal to ℑ*(α*_far_*) *= 0.00075 and ℑ*(α*_near_*) *= 0.206. Equation 4 has been calculated for the six values of *μ*_e _due to progressive addition of SWCNHs: results for ℑ*(α*_far_*) *were almost identical to the values obtained using Equation 5 in the calculation, while for ℑ*(α*_near_*)*, we obtained a value range from 0.180 to 0.198 at *λ *= 600 nm. In the worst case, this difference could lead to relative errors of 10% to 15% in the albedo, which is acceptable in the framework of the proposed estimation method aimed to assess a higher boundary.

Assumptions (3) and (4) have been investigated by means of Monte Carlo simulations. We calculated the transmittance for different values of *τ*_e _ranging from 0.2 to 2.5, using a code for photon migration through a three-layer slab in which multiple scattering and internal reflections are taken into account [16]. For the turbid medium, we assumed the Rayleigh scattering function and *ω *= 0.1, and we considered the same geometry as the experiment. The results do not differ if assumptions either (3), (4), or both are made.

As for hypotheses (5) and (6), they do not appreciably affect the results. In fact, numerical investigations showed that in all our experiments, $\varepsilon_{\text{e}}\rho L\omega ℑ\left(\alpha \right)$ was always smaller than 0.025 (in fact, in writing $\text{ln}\left[\varepsilon_{\text{e}}\rho L\omega ℑ\left(\alpha \right)+1\right]=\varepsilon_{\text{e}}\rho L\omega ℑ\left(\alpha \right)$, we made the hypothesis $\varepsilon_{\text{e}}rLwℑ\left(\alpha \right)<<\text{1}$ and that the difference between *ε*_e _and *ε*_e meas_*(α*_far_*) *was smaller than 0.1%.

## Results and discussion

**Figure 2:**
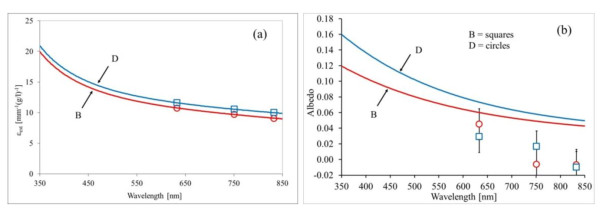
**Spectral extinction coefficient (a) and fitted albedo values (b), with error bars (gray bars)**. Hollow squares (B) and hollow circles (D) in both images show the results of monochromatic measurements performed with the multidistance method. D and B label dahlia-like and bud-like SWCNH morphologies, respectively.

The spectral extinction coefficient of an undiluted suspension of SWCNHs is calculated for the two considered morphologies as described above. The results are depicted in Figure 2a. In the same figure, we also show (hollow square and hollow circle symbols) the values of *ε*_e _previously measured at three fixed wavelengths with a different experimental apparatus based on laser sources. The details of the setup, method, and results are reported elsewhere [12]. The agreement between spectrophotometric and monochromatic methods is very good. From Figure 2a, we can note that for both suspensions, the shorter is the wavelength, the higher is the extinction coefficient, with a small difference between the two considered morphologies.

The results for the albedo are reported in Figure 2b. In the same figure, we also show the values obtained at *λ *= 633, 751, and 833 nm with the different experimental techniques cited above (multidistance measurement in an infinite diffusive medium [8,12]) for comparison. Also, in the case of albedo, the agreement between the two considered experimental methods is satisfactory, taking into account that the spectrophotometric method provides an upper limit for albedo within the Rayleigh hypothesis as discussed. It should be emphasized that the spectrophotometric and multidistance techniques are completely different. The latter [17] consists of adding small amounts of the suspension under investigation to a previously calibrated diffusive medium and of performing, within the resulting liquid, multidistance measurements of fluence rate at fixed distances from an optical fiber radiating isotropically. This technique is based on the solution of diffusion equation for an infinite medium with known scattering properties, in which a small amount of the medium under investigation is introduced in several steps. On the contrary, the spectrophotometric technique needs no reference suspension and no previous calibration even if it requires some hypothesis about the scattering function.

The proposed technique could be of interest for all applications requiring the characterization of light-scattering properties of suspensions, like solar energy exploitation and biological tissue optics. As for this latter application, the results of our investigations on SWCNH suspensions show that they can be promising, especially when they are compared to absorber materials currently used in liquid phantoms, i.e., Indian inks [17]. It should be emphasized that literature values of scattering albedo for Indian inks at NIR wavelengths lie between 10% and 16%. For this reason, the ink behavior is far from that of an ideal absorber whose properties are a high absorption coefficient and a 0% scattering. In this framework, SWCNH-based nanofluids, thanks to their low albedo values, have the potential to be an excellent absorbing standard for biological tissue optics.

## Conclusions

In this paper, a simple spectrophotometric method is applied to the calculation of the spectral scattering albedo of SWCNHs. The results show a satisfactory agreement with those obtained from monochromatic measurements reported in the literature and performed with a different technique. Thus, the present paper confirms that SWCNHs have a very low scattering albedo (not higher than 5% for red and NIR wavelengths) as compared to Indian inks (about 10% to 16% scattering). Moreover, the scattering behavior of SWCNHs shows little dependence on the nanohorn morphology (dahlia-like or bud-like). These results are very interesting to assess the SWCNH potential in different applications, like for example, their use as absorbing standard in biological tissue-simulating phantoms, for the calibration of noninvasive diagnostic optical devices. Moreover, SWCNH suspensions show good stability properties as confirmed by preliminary measurements we performed over a period of 1 year.

## Competing interests

The authors declare that they have no competing interests.

## Authors' contributions

LM, ES, AG, and PDN performed the spectrophotometric measurements and data analysis. FM and GZ were involved in the theoretical modeling. All authors read and approved the final manuscript.
